# Prediction of the Active Components and Possible Targets of Xanthii Fructus Based on Network Pharmacology for Use in Chronic Rhinosinusitis

**DOI:** 10.1155/2022/4473231

**Published:** 2022-02-03

**Authors:** Shun Ding, Tingting Duan, Zhengyang Xu, Dongqin Qiu, Jingren Yan, Zhonglin Mu

**Affiliations:** Department of Otolaryngology, Head and Neck Surgery, The First Affiliated Hospital, Hainan Medical University, Haikou 570102, China

## Abstract

Chronic rhinosinusitis (CRS) is a complex condition brought on for many reasons, and its prevalence is rising gradually around the world. Xanthii Fructus (XF) has been used in the treatment of CRS for decades and is effective. The chemical and pharmacological profiles of XF, on the other hand, are still unknown and need to be clarified. The potential mechanisms of XF in CRS treatment were investigated using a network pharmacology approach in this study. OB and DL were in charge of screening the bioactive components in XF and drug-likeness. TCMSP and PubChem databases were used to identify prospective XF proteins, whereas GeneCards and the DisGeNET database were used to identify potential CRS genes. An interactive network of XF and CRS is built using the STRING database based on common goals identified by the online tool Venny. Cytoscape was used to visualize the topological characteristics of nodes, while the biological function pathways were identified by GO Knowledge Base, KEGG. There were 26 bioactive components and 115 potential targets in XF that bind to CRS or are considered therapeutically relevant. Five significant signaling pathways have been found for CRS by the pathway analysis including the HIF-1 signaling pathway, TNF signaling pathway, Toll-like receptor signaling pathway, NOD-like receptor signaling pathway, and PI3K-Akt signaling pathway. We simultaneously confirmed that the PI3K-Akt pathway promotes the development of CRS. Finally, this study took a holistic approach to the pharmacological actions and molecular mechanisms of XF in the treatment of CRS. TNF, INS, CCL2, CXCL8, IL-10, VEGFA, and IL-6 have all been identified as potential targets for anti-inflammatory and immune-boosting effects. This network pharmacology prediction could be useful in manifesting the molecular mechanisms of the Chinese herbal compound XF for CRS.

## 1. Introduction

Chronic rhinosinusitis (CRS) is an inflammation of the nasal and sinus, which has a complex etiology including immunological and epithelial barrier components affected by the microbiota, environment, and genetic factors, while the course of CRS is more than 12 weeks [1]. CRS has many chronic symptoms including congestion, stuffiness, nasal discharge, discomfort or face pressure, loss of smell (anosmia), cough, and exhaustion [2]. According to current statistics, the prevalence of CRS, as defined by the European Position Paper on Sinusitis and Nasal Polyps (EPOS) symptoms, is 8% in China [3], while 11% in Europe and 12% in the United States [4]. The therapy, which includes rationally using systemic corticosteroids, specific immunotherapy, surgical approach, antibiotic adequacy, and administration of recently authorized biologics, is still up for discussion [5]. It not only inevitably prolongs the treatment, but also consumes significant direct medical expenditures, renders the patient incompetent, and causes several indirect costs to society [6].

People are progressively pursuing health, while the notion of healthy living changed dramatically. Traditional Chinese medicine's (TCM)' distinctive effects not only evoked the interest of domestic intellectuals but also had stirred up the interest of the worldwide community [7]. The fruit of *Xanthium* sibiricum Patrin is called Xanthii Fructus (XF). It' has been used as an herbal medicine for thousands of years to treat quite a few diseases, such as nasal blockage, sinusitis with nasal discharge, nasal discharge, urticaria with itching, and rhinitis with muscle spasms caused by wind and moisture [8]. Huang et al. [9] found that the extracts of XF have the functions for antioxidant, antinociceptive, and anti-inflammatory properties in the carrageenan-induced hind paw edema model, while An et al. [10] discovered that XF extracts could suppress phlegmonous of mice peritoneal macrophages stimulated by lipopolysaccharide. Furthermore, Song et al. [11] discovered that XF extracts could protect the injury of pancreatic beta cells by inhibiting nuclear factor kappa B activity, but the effect of XF on the therapy of CRS has yet to be confirmed.

**Figure 1:**
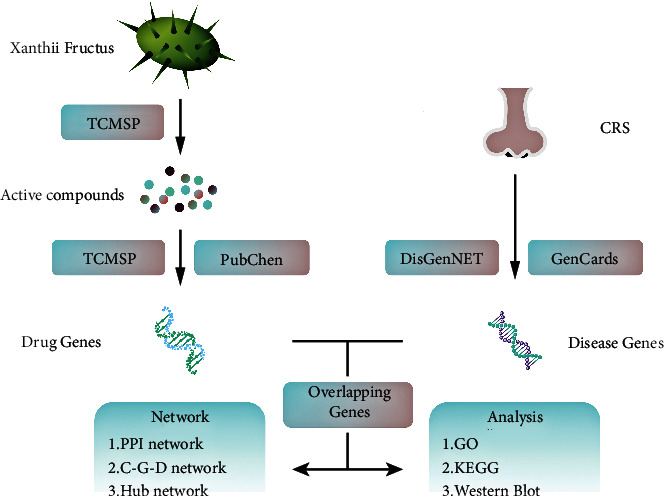
Scheme of this study.

In this article, the studies related to XF for the treatment of CRS were carried out by the network pharmacology approach. The target proteins were selected among the ingredients of XF and the treatment of CRS, and the core was built by compound-gene-disease (C-G-D) network between XF and CRS. Using overlapping genes, protein-protein interaction (PPI) networks were created. Hub genes were chosen from Gene Ontology (GO) and Kyoto Encyclopedia of Genes and Genomes (KEGG) pathway enrichment analysis. The goal of this research was to find a possible therapeutic target for XF in the treatment of CRS and to create the groundwork for further research into the pharmacological mechanism of XF (see Figure 1).

## 2. Materials and Methods

### 2.1. XF Bioactive Ingredients

The traditional Chinese medicine systems pharmacology (TCMSP) database (https://tcmsp-e.com/) was used to find all of the chemicals in XF [12]. The most essential restrictions in the screening of active components were absorption, distribution, metabolism, and excretion (ADME) of pharmacological substances. The most important pharmacokinetic characteristics in ADME processes are oral bioavailability (OB) and drug-likeness (DL) [13]. The rate and degree of absorption of oral drugs from the gastrointestinal tract into the body's circulation are known as OB. Herbal ingredients that are structurally similar to known drugs are called DL. Compounds OB ≥ 20% and DL ≥ 0.1 were evaluated as bioactive components in this investigation.

### 2.2. The Acquisition of Gene Targets for XF

Compounds influence biological activity at both the transcriptional and protein levels by binding to certain molecular targets. The chemical-target interaction was necessary for elucidating the mode of compound action, which was obtained from the TCMSP database (http://www.tcmspw.com/tcmsp.php) and the PubChem database (https://pubchem.ncbi.nlm.nih.gov/) [14]. The PubChem database was used to capture the top 100 most frequent target genes for each active compound through the “Chemical-Gene Co-Occurrences in Literature” module. The standardization of gene names is according to the UniProt database (http://www.uniprot.org/) [15]. The targets of XF were obtained after removing the duplicate targets.

### 2.3. Collecting Disease Targets

To discover CRS-related targets, we searched the GeneCards database (https://www.genecards.org/) and DisGeNET database (http://www.disgenet.org/) using the keyword “chronic rhinosinusitis.” The GeneCards database contains detailed information on human genes, which have been annotated [16]. The DisGeNET database is a public-access discovery platform that holds one of the largest databases of genes and variations connected to human disorders [17]. With the online tool Venny 2.1 (http://bioinfogp.cnb.csic.es/tools/venny/), the intersection between the probable target genes of XF and CRS-related targets was determined, and these were considered potential targets in XF for the treatment of CRS, which was visualized with a Venn diagram.

### 2.4. Compound-Gene-Disease (C-G-D) Network Construction

To get the overlapping genes, we took the target genes of XF and the therapeutic targets of CRS from the above four databases. Following that, we combined the information from the component, genetic, and diseases while we construct C-G-D using the Cytoscape software (v.3.6.0, https://cytoscape.org/) [18].

### 2.5. Network Analysis of Protein-Protein Interactions (PPI)

For PPI network research, the common genes of XF and CRS have been uploaded to the STRING database. PPI network visualization was established on associated proteins with a confidence score ≥0.4 (https://string-db.org/, version 11.0) [19]. In the Cytoscape software, the generated PPI network was exhibited, which was based on the STRING database' information. The Cytoscape CytoHubba plug-in was used to calculate the top ten genes that were potential hub targets with a high degree of accuracy [20]. The network's most topologically important node has betweenness centrality, the highest degree, and closeness centrality.

### 2.6. Analyses of GO and KEGG Pathways

GO Knowledge Base can help us learn about gene functions such as acellular components (CCs), biological processes (BPs), and molecular functions (MFs) [21], and KEGG was a database that provided access to gene function and genomic data at a high level [22]. The GO and KEGG pathway enrichment investigations were carried out using DAVID (https://david.ncifcrf.gov/), a collection of gene annotation and analysis resources [23]. The clusterProfiler package in *R* chooses the top 10 BP, CC, and MF GO keywords, as well as the top 20 KEGG pathways, bubble diagrams for functional annotation clustering to structure enrichment analysis, based on a high count and *p* < 0.05 [24]. Enrichment analysis could help researchers learn more about the biological activities and probable processes of the XF targets in CRS.

### 2.7. Animals, Drugs, and Reagents

Thirty healthy rabbits at the age of 3 months, weighing 1.6-2.5 kg, were purchased from Fengdong New City Experimental Animal Farm, Xi'an New District (Certificate No. (SCXK(Shan)2017-002). Animals were kept in the SPF animal laboratory at room temperature of 20-25°C, relative humidity of 45%-50%, normal light, and free access to food and water. The maker of XF granules was Guangdong Yifang Traditional Chinese Medicine Granules Co., Ltd., which was obtained from Hainan Provincial Hospital of Traditional Chinese Medicine. The experimental procedure adheres to applicable animal ethics guidelines. 2) Primary antibodies against PI3K, AKT, and *β*-actin were acquired from Abcam, while secondary antibodies labeled with horseradish peroxidase were purchased from Shanghai Biyuntian Co.

### 2.8. Bacteria

Bacteria were obtained from maxillary sinus puncture rinses of outpatients with CRS, and a single strain of *Staphylococcus aureus* was inoculated in the culture medium and placed in an incubator for 24 h. Colonies were collected and diluted with saline into a bacterial suspension of 1 ms unit and set aside.

### 2.9. Molding and Drug Delivery

Thirty rabbits were divided into three groups at random: the control group, the model, and the XF group, with 10 rabbits in each group. A longitudinal incision was made along the midline of the nasal dorsum, and the subcutaneous tissue and periosteum of the anterior maxillary sinuses were separated bilaterally. The maxillary sinus puncture needle was used to drill a small hole of 1.5 mm in diameter in the depression of the round hole of the anterior maxillary sinus wall; meanwhile, a few cotton wool was placed in the sinus opening and sinus cavity. Then, 1 mL (3∗108(CFU)/mL) of *Staphylococcus aureus* suspension was injected, the surgical wound was disinfected with iodophor, and the periosteum and skin were sutured layer by layer. The surgical procedure was the same for the XF group. The normal control group did not require any treatment. The duration of modeling was 42 d. From the seventh week, the XF group was treated with 350 mg/(Kg.d) of gavage, and the rest of the groups were treated with an equal volume of saline, and the duration of treatment for each group was 14 d. Afterward, the nasal tissue was removed by execution.

### 2.10. Western Blot for PI3K and AKT Expression

Nasal tissues were extracted in each group, and extracted total protein was used to detect PI3K and AKT expression after grinding and milling. The concentration of each set of proteins was determined using the BCA technique, and the proteins were separated using SDS-PAGE electrophoresis. The proteins were transferred to the PVDF membrane, followed by 5% skim milk closure for 2 h. Anti-rabbit PI3K protein (1 : 1000) antibody and anti-rabbit AKT protein (1 : 2000) antibody were added, kept warm at 4 °C, and incubated overnight, the primary antibody was washed away by TBST, secondary antibody (1 : 5000) was added at room temperature 1 h, TBST washed off the secondary antibody and imaged with ECL chemiluminescent solution for 3 min, and the target bands/corresponding relative expression levels were calculated using Image*J* software after the Western blot bands were evaluated.

### 2.11. Statistical Analysis

Based on the characteristics of comparison, the statistical analyses were conducted with Excel 2016 and GraphPad Prism version 5.0 software, using an unpaired *t*-test with *p* < 0.05.

## 3. Results and Discussion

### 3.1. Screening of Active Compounds

Based on the OB and DL values, the TCMSP database was searched to locate 26 active components. The active compounds were supplanted by numbers to facilitate future data analysis (see Table 1).

### 3.2. Potential Targets of XF for CRS

**Figure 2:**
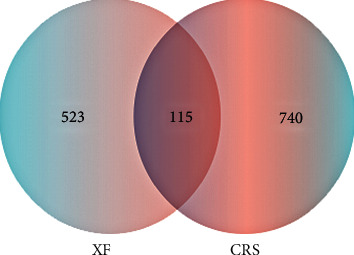
Drugs and disease targets are depicted in a Venn diagram.

The TCMSP database and the PubChem database yielded 1603 targets among the 26 candidate components, after removing the overlapping target, and there were a total of 638 ingredient targets in XF. Since removing duplicates from the GeneCards and DisGeNET databases, 855 genes involved with CRS were discovered. After that, using the Venny 2.1, the intersection of 638 XF targets and 855 CRS-related targets was determined. As a consequence, Venn diagrams revealed 115 potential genes coupled to both CRS and XF (see Figure 2).

### 3.3. XF Compound-Target Network

**Figure 3:**
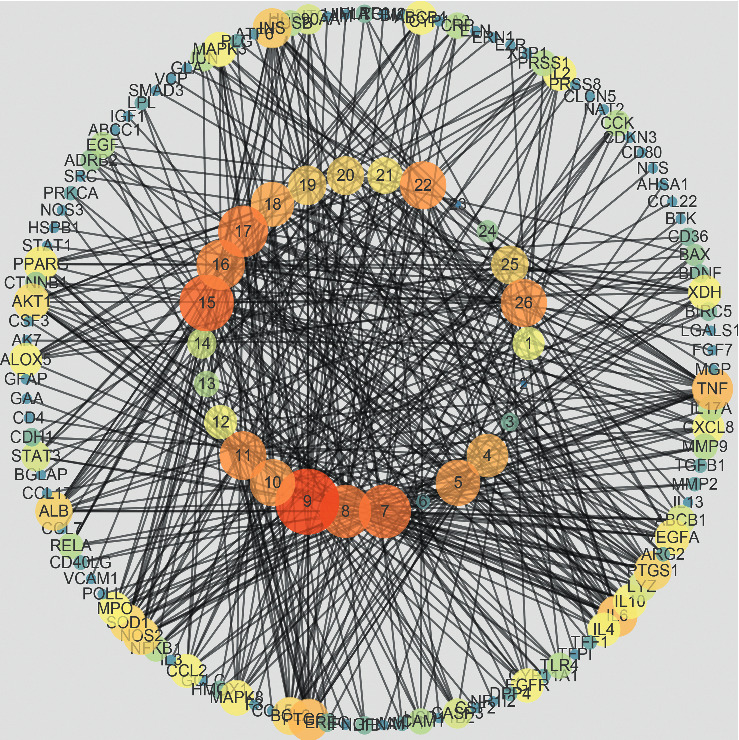
C-G-D network of XF. The inner circle represents the compounds, and the outer circle represents the targets (determine the size of a single circle by the level of degree).

Because Chinese medicinal formulae have multiple pharmacological impacts by interacting with various targets, network analysis is a useful method for studying the underlying mechanisms of Chinese medicinal formulae on complicated disorders. The construction C-G-D network of XF on CRS was erected using Cytoscape 3.6.0 (see Figure 3). The network had 141 nodes and 473 edges, according to network analysis in Cytoscape 3.6.0. Multiple CRS targets were controlled by a single active compound. These findings could lead to new mechanisms for treating CRS with XF.

### 3.4. The PPI Network of Target Proteins Was Built and Analyzed

**Figure 4:**
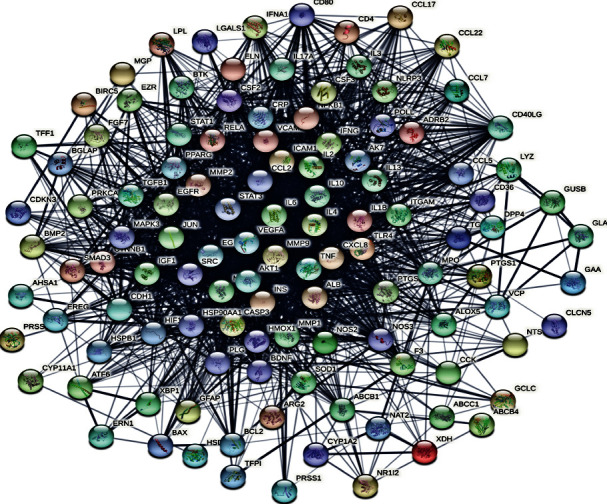
Interaction network of common target proteins.

**Figure 5:**
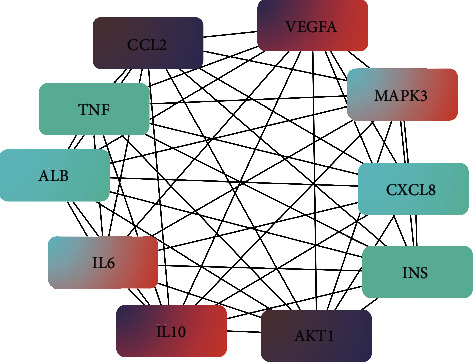
MCC algorithm was used to analyze the top 10 hub genes' network of XF for CRS treatment.

Increasing evidence suggests that diseases are caused by interactions among multiple targets rather than by a single gene. Potential genes were entered into the STRING database to build a PPI network to further elaborate the molecular mechanisms of XF's pharmacological actions on CRS. Proteins and protein-protein interactions are represented by the network's nodes and edges. After hiding disconnected nodes, the network was established with 115 nodes and 2041 edges in this study (see Figure 4). The average node degree was 35.5, and the average local clustering coefficient was 0.715. Potential hub genes of XF for treating CRS, which include TNF, MAPK3, ALB, INS, CCL2, CXCL8, IL-10, AKT1, VEGFA, and IL-6, were discovered by the CytoHubba plug-in with a degree based on their degree (see Figure 5). The network features of potential hub targets are summarized in Table 2 (see Table 2).

### 3.5. GO Enrichment Analysis

**Figure 6:**
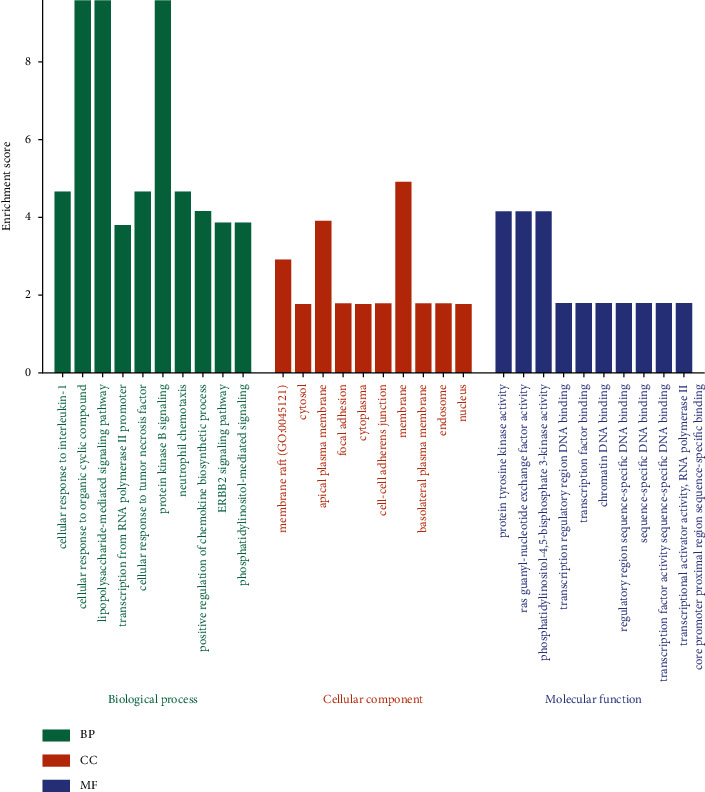
Evaluation of targets based on the GO. Top 10 of BP, CC, and MF term enrichment.

The above common targets were entered into DAVID for GO enrichment analysis to show the numerous biological functions of probable targets in CRS following XF therapy. The biological process (BP) was significantly enriched in the cellular response to interleukin-1 (GO:0071347), cellular response to organic cyclic compound (GO:0071407), lipopolysaccharide-mediated signaling pathway (GO:0031663), positive regulation of pri-miRNA transcription from RNA polymerase II promoter (GO:1902895), and cellular response to tumor necrosis (GO:0071407), cellular components (CCs) in membrane raft (GO:0045121), cytosol (GO:0005829), apical plasma membrane (GO:0016324), focal adhesion (GO:0005925), and cytoplasm (GO:0005737), molecular function (MF) in protein tyrosine kinase activity (GO:0004713), Ras guanyl nucleotide exchange factor activity (GO:0005088), phosphatidylinositol-4,5-bisphosphate 3-kinase activity (GO:0046934), transcription regulatory region DNA binding (GO:0044212), and transcription factor binding (GO:0008134) (see Figure 6).

### 3.6. KEGG Enrichment Analysis

**Figure 7:**
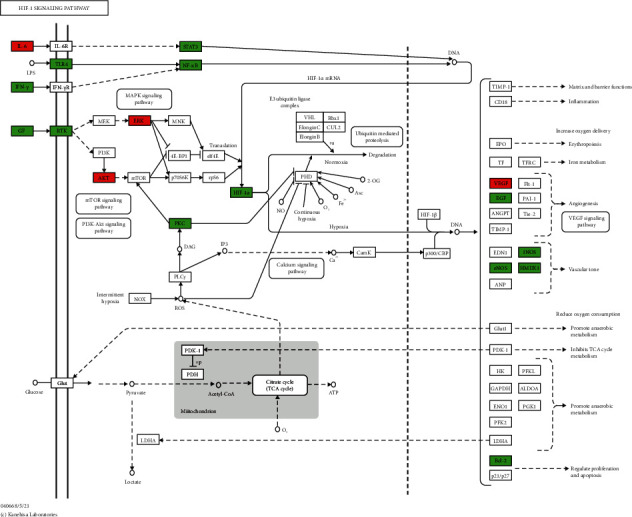
HIF-1 signaling pathway. The red rectangle represents the Hub genes, and the green represents the predicted target genes.

**Figure 8:**
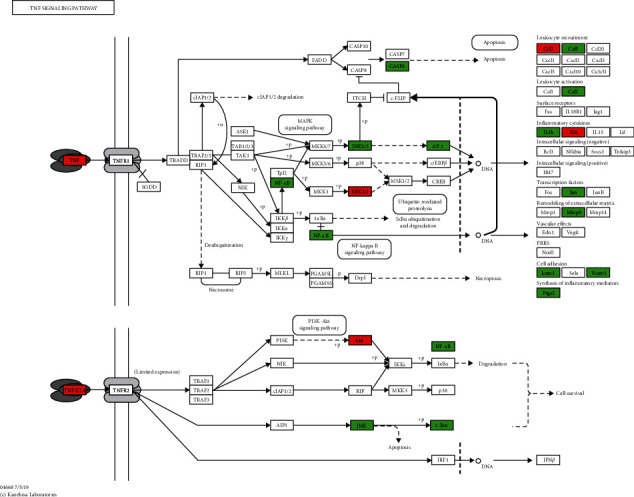
TNF signaling pathway. The red rectangle represents the Hub genes, and the green represents the predicted target genes.

**Figure 9:**
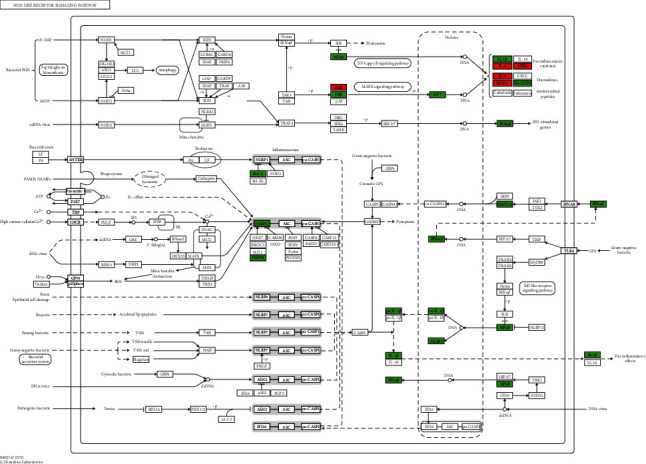
NOD-like receptor signaling pathway. The red rectangle represents the Hub genes, and the green represents the predicted target genes.

**Figure 10:**
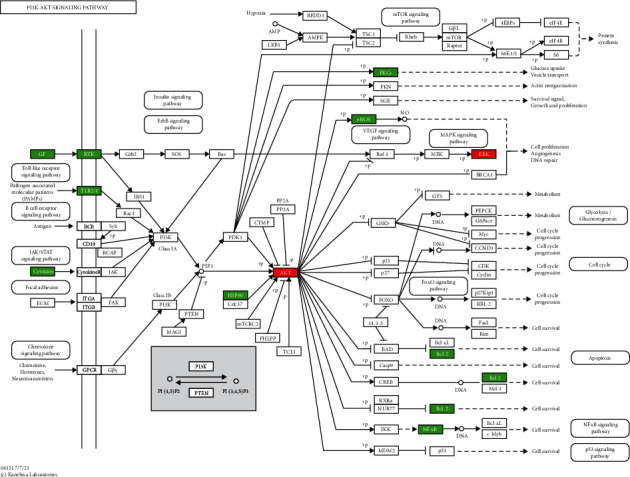
PI3K-Akt signaling pathway. The red rectangle represents the Hub genes, and the green represents the predicted target genes.

**Figure 11:**
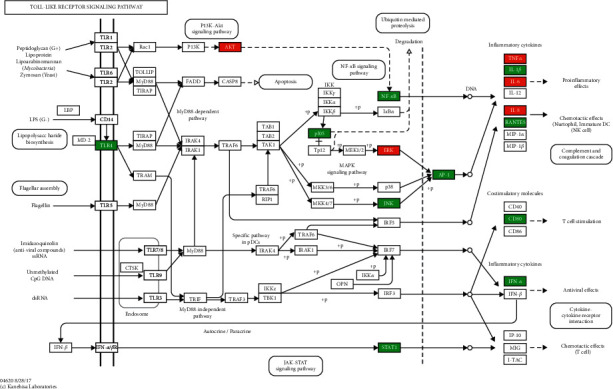
Toll-like receptor signaling pathway. The red rectangle represents the Hub genes, and the green represents the predicted target genes.

**Figure 12:**
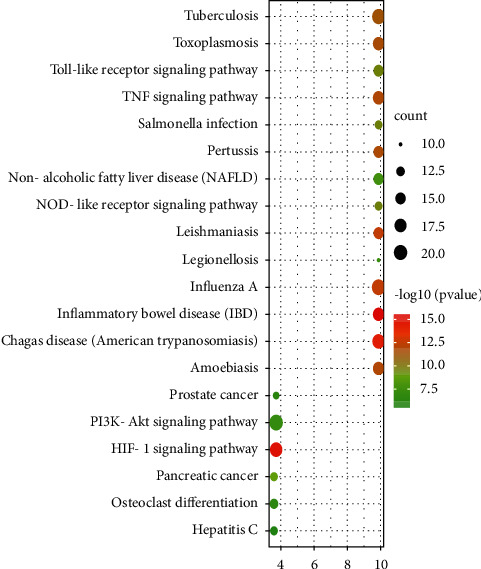
KEGG analysis of the top 20 pathways. The size of each dot equates to the number of genes cataloged in the entry, and the color of each dot relates to the corrected *p* value.

The 115 predicted target genes were approached into DAVID for KEGG pathway enrichment analysis to better understand the key signaling pathways of XF in the therapies of CRS. The top 20 pathways were evaluated using a combination of enrichment scores and *P* values (see Table 3), including the HIF-1 signaling pathway (hsa04066), TNF signaling pathway (hsa04668), NOD-like receptor signaling pathway (hsa04621), PI3K-Akt signaling pathway (hsa04151), and Toll-like receptor signaling pathway (hsa04620) (see Figures 7–11). The Omcishare website was used to display the KEGG pathways based on their matching counts (https://www.omicshare.com/) (see Figure 12).

### 3.7. The PI3K/AKT Signaling Pathways Are Regulated by XF Treatment in Nasal Mucosa Tissue

**Figure 13:**
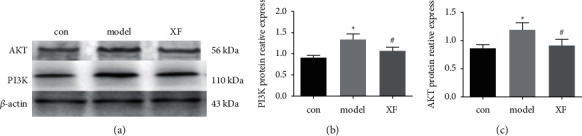
CRS anti-inflammatory activity is regulated by XF via the PI3K/AKT signaling pathways. (a) PI3K and AKT expression levels in different groups. (b) PI3K protein reactive expression. (c) AKT protein reactive expression. ^∗^*p* < 0.05 Vs control, ^#^*p* < 0.05 Vs model.

PI3K/Akt signaling pathway is widely prevalent in cells and causes inflammatory responses. To investigate the anti-inflammatory effects of XF on CRS, we looked at changes in the PI3K/AKT signaling pathways in nasal mucosa tissue. PI3K and AKT are significantly elevated in CRS. We found that XF reduced the expression of PI3K and AKT in nasal mucosa tissue. These findings suggest that XF treatment can control CRS anti-inflammatory activity by inhibiting the PI3K/AKT signaling pathways (see Figure 13).

## 4. Discussion

Mucosal inflammation is the common thread that runs through CRS, corticosteroids, antibiotics, and surgery that are the most common treatments for CRS. However, the symptoms of CRS patients vary and do not meet the needs of individualized clinical treatment [25]. TCM is a vital component of today's healthcare system. It aims to strengthen the physical fitness of the patient population through the use of herbal formulas (Fang-Ji in Mandarin), which is often made up of two or maybe more medicinal plants and can be used to treat illnesses in a systematic manner [26]. Natural herbs and herbal substances grouped into a recipe have been shown to have potential interaction effects. Mutual benefit, mutual help, mutual restraint, and mutual enmity are instances of these [27]. Herbal medications from China may have a variety of therapeutic effects, including multiple targets and pathways, due to their multicomponent compositions, which may be beneficial in the treatment of CRS [28]. In the treatment of CRS, XF has demonstrated promising results. However, the underlying processes of XF in the treatment of CRS are unknown and must be explored through a thorough molecular investigation. Network pharmacology can build complicated interaction communication that relies on target molecules, bioactive compounds, and biological functions, according to theories of systems biology, molecular biology, and pharmacology, and may make clear the potential molecular pathways of complex and difficult Chinese medicine (CM) formulae in diseases. In this work, the above approach was employed to shed light on the hidden pharmacological processes of XF in CRS therapy. Pharmacokinetically active components were found to be consumed and distributed in the body in XF with OB ≥ 20% and DL ≥ 0.1. As a result, the 26 bioactive components and 115 possible targets of XF for CRS treatment have been anticipated, potentially revealing the pharmacological processes of XF. The 10 hub targets were TNF, MAPK3, ALB, INS, CASP3, MMP9, EFG, AKT1, VEGFA, and IL-6.

According to the literature, anatomical blockage of the stomatal complicated, impaired respiratory system, chronic sinusitis, microbes, biofilms, immune disorder, deficient epithelial defense, biological factors, and environmental factors are all factors that influence the prognosis of CRS [29]. Linoleic acid, which is closely related to CRS treatment, was the most significant compound in this study. In inflammatory diseases, fatty acid metabolism has begun to play a role in regulating immune responses [30]. Polyunsaturated fatty acids have previously been shown to affect T-cell functions by inhibiting proliferation and activation, as well as suppressing mast cell activation and secretion [31]. Linoleic acid is essential in activating both autophagy and antioxidation in a synergistic feedback loop, which helps to prevent and treat a variety of inflammatory diseases [32]. As a result, we have reason to believe that linoleic acid is important in the CRS. *β*-Patchoulene is a substance that has long been used in traditional Chinese medicine to treat inflammatory diseases, while inhibiting ear edema induced by xylene and paw edema induced by carrageenan and suppressing the increase in vascular permeability induced by acetic acid in a dose-dependent manner. In edema paws, *β*-Patchoulene was also shown to reduce malondialdehyde (MDA) levels and myeloperoxidase (MPO) activity. Furthermore, in a dose-dependent way, carrageenan-induced production of several pro-inflammatory cytokines, such as TNF, IL-1, IL-6, PGE2, and NO, was decreased in mice pretreated with *β*-patchoulene [33]. Inflammatory factors on the cell membrane of CRS are interfered with *β*-sitosterol. *β*-sitosterol inhibited caspase-1 activation and decreased the expression of NLRP3, a key component of the NLRP3 inflammasome. NF–B was partially suppressed in macrophages [34]. By blocking p-IKB activation and downregulating the expression of NF-kBp65, stigmasterol lowers the expression of pro-inflammatory mediators (TNF, IL-6, IL-1, iNOS, and COX-2) while raising the expression of anti-inflammatory cytokine (IL-10) (p38MAPK in anti-inflammatory) [35]. With all of these studies combined, a network pharmacology approach predicts that XF may improve the cure rate through anti-inflammatory effects.

Several genes implicated in the occurrence and development of CRS were found among some of the top 10 hub targets, and they play a critical role in the therapeutic value of XF for CRS treatment. In CRS, the pro-inflammatory effects of IL-6 trans-signaling have been connected to the transition from acute innate immune responses to adaptive chronic inflammatory reactions [36]. IL-6 inhibits neutrophil recruitment and enhances granulocyte death during innate immune responses. In adaptive immune responses, IL-6 trans-signaling is required for T-cell recruitment and survival [37]. TNF-*α* is a kind of TNF. In CRS, TNF-*α* causes small proline-rich protein dysregulation. This may have resulted in a breakdown of the epithelial barrier. VEGFA can promote tissue remodeling and angiogenesis in CRS. CCL2 is a chemokine that plays a pivotal role in the inflammatory response. It acts as a chemoattractant for monocytes and macrophages and is involved in a variety of immune processes [38]. CXCL8 are primarily promoters that play a role in a variety of tissue inflammation and angiogenesis processes [39]. By attaching to the leukocyte receptor, CXCL8 controls the migration of circulating leukocytes to inflammation or damage sites [40]. Rudack et al. established in their CRS investigation that CXCL8 can be produced by activating nuclear factor kappa B (NF–B) signaling after stimulation of the protease-activated receptor 2 (PAR-2) [41]. In CRS, anti-inflammatory cytokine IL-10 is essential for limiting and eventually terminating immune and inflammatory responses [42]. INS can reduce inflammatory factors and promote the healing of nasal epithelium [43]. Based on the above results, XF mainly regulates nasal inflammation and the immune environment to achieve the healing effect.

As shown in a KEGG pathway enrichment analysis, many signaling pathways were utilized in the care of CRS with XF in this study. XF-associated hub targets were found in the HIF-1 signaling pathway, TNF signaling pathway, Toll-like receptor signaling pathway, NOD-like receptor signaling pathway, and PI3K-Akt signaling pathway, which are all closely relevant to CRS. The HIF-1 signaling pathway is a multifunctional angiogenic regulator that promotes endothelial cell proliferation and migration, while also increasing vascular permeability [44], plasma proteins, inflammatory mediators, and inflammatory cells leak from the nasal mucosa into the extravascular space of the airway [45]. HIF-1 also promotes pro-inflammatory cytokine production and immune cell adhesion, as well as aiding inflammatory cell energy metabolism [46]. All of these functions are necessary for immune and inflammatory responses to occur. Toll-like receptors (TLRs) are the most common pattern recognition receptors (PRRs), and they play an important role in recognizing different microbial components and initiating a signaling cascade that activates immune cells directly [47]. Downregulation of a TLR-mediated signaling pathway is a feature of chronic rhinosinusitis without nasal polyps (CRSsNP), and this innate immune system deficiency may contribute to CRSsNP's inflammatory process. The intracellular nucleotide-binding oligomerization domain-containing protein 2 (Nod2)-like receptor detects a small portion of muramyl dipeptide peptidoglycans found in the bacterial wall (MDP) [48]. RIPK2, a serine/threonine kinase, is recruited by Nod2, and NF-*κ*B is discharged from its inhibitors by RIPK2, which activates a kinase complex. NF-*κ*BIA is the most well-studied NF-*κ*B inhibitor, and it works by sequestering NF-*κ*B in the cytoplasm and blocking it from binding to DNA, therefore inhibiting NF–B-dependent transcription. Nod2 activation in CRS causes the breakdown of NF-*κ*BIA and subsequent release of NF-*κ*B, which translocates into the nucleus to transcribe pro-inflammatory genes [49]. When compared to the normal human nasal mucosa, TNF was significantly higher in chronic rhinosinusitis with polyp patients. After LPS stimulation, TNF expression levels rose in a dose- and time-dependent manner. In CRS with NP, TNF plays a role in regulating the body's inflammatory response [50], which can also kill transformed cells and certain virus-infected cells, and not only has no damaging effect on normal cells but, on the contrary, can stimulate their production [51]. A large number of hub genes were enriched in the above-mentioned pathways. In conclusion, XF may have therapeutic effects in CRS, primarily through anti-inflammatory and immune-boosting effects, which is in line with recent CRS research findings.

In rhinology, CRS is a prevalent condition, with chronic sinusitis without nasal polyps (CRSsNP) accounting for roughly 60% of cases. CRSsNP typically begins as a bacterial infection that progresses to chronic inflammation if left untreated [52]. In the sinuses of adults with CRS, Gram-positive bacteria such as coagulase-negative staphylococci (such as *Staphylococcus aureus* and *Streptococcus* viridans) and Gram-negative enterobacteria have been found. Mucosal inflammation with neutrophil and monocyte infiltration, extracellular matrix reconstruction, cupped cell proliferation, and tissue necrosis are all symptoms of bacterial infection [53, 54]. For the following research, a rabbit model of bacterial CRS mimicking CRSsNP was established. Bacteria are frequently employed to create CRS models for research in both national and international investigations. The PI3K/Akt signaling pathway is widely prevalent in cells and is important in cell cycle and apoptosis regulation, among other things. Sarkar et al. [55] found that activating the PI3K/Akt signaling system causes an inflammatory response, while inhibiting the signaling pathway reduces the inflammatory response considerably [56]. As a result, examining the interplay between inflammatory cytokines and the PI3K/Akt signaling pathway can aid in the exploration and discovery of the process of CRS incidence and progression. In the study, PI3K and AKT are significantly elevated in CRS. We found that XF reduced the expression of PI3K and AKT in nasal mucosa tissue. These findings suggest that XF treatment can control CRS anti-inflammatory activity by inhibiting the PI3K/AKT signaling pathways.

This study, however, had some limitations. For example, it was unclear whether the pathways studied in this study were downregulated or upregulated. At the same time, we focus on the animal model of CRSsNP and use this model to demonstrate that XF has a regulatory role in CRSsNP through the PI3K/AKT pathway. However, the pathogenesis of chronic rhinosinusitis with nasal polyps (CRSwNP) is somewhat different from that of CRSsNP, so this model cannot be used to show the same role in CRSwNP. In addition, the experimental validation of this study was mainly at the animal level and did not involve human specimens, and it remains to be studied whether XF plays the same role in the treatment of CRS in humans as it does in animal studies. The network pharmacology approach identified potential targets of XF in CRS treatment, but these are theoretical predictions for now, which need to be confirmed in clinical and cellular or animal studies.

## Figures and Tables

**Table 1:** 26 compounds from XF, along with their predicted OB, DL, and substitute number.

Mol ID	Molecule name	OB (%)	DL	Substitute number
MOL011676	Carboxyatractyloside	39.9686979	0.47025	1
MOL011678	(3S,8S,9S,10 R,13R,14S,17R)-17-[(1S,4 R)-4-ethyl-1,5-dimethylhexyl]-10,13-dimethyl-2,3,4,7,8,9,11,12,14,15,16,17-dodecahydro-1h-cyclopenta[a]phenanthren-3-ol	36.91390583	0.75147	2
MOL000131	EIC	41.90443602	0.14347	3
MOL002573	*β*-Patchoulene	50.68856541	0.10733	4
MOL000357	Sitogluside	20.63193686	0.6241	5
MOL003574	*α*-Gurjunene	52.57024488	0.10394	6
MOL000359	Sitosterol	36.91390583	0.7512	7
MOL000471	Aloe emodin	83.37963699	0.2409	8
MOL000472	Emodin	24.39832432	0.23916	9
MOL000675	Oleic acid	33.12836481	0.14243	10
MOL007326	Cynarin(e)	31.75850133	0.67849	11
MOL000011	(2R,3 R)-3-(4-Hydroxy-3-methoxy-phenyl)-5-methoxy-2-methylol-2,3-dihydropyrano[5,6-h] [1, 4]benzodioxin-9-one	68.82559903	0.66236	12
MOL000208	()-Aromadendrene	55.7416731	0.10418	13
MOL000266	Beta-cubebene	32.81330687	0.10858	14
MOL000358	Beta-sitosterol	36.91390583	0.75123	15
MOL000432	Linolenic acid	45.00906591	0.14709	16
MOL000449	Stigmasterol	43.82985158	0.75665	17
MOL000474	(-)-Epoxy caryophyllene	35.93684943	0.12925	18
MOL000749	Linoleic	41.90443602	0.14468	19
MOL001442	Phytol	33.82439209	0.13342	20
MOL001755	24-Ethylcholest-4-en-3-one	36.08361164	0.75703	21
MOL002683	Ligla	45.00906591	0.14562	22
MOL005961	10,13-Octadecadienoic acid, methyl ester	41.93435814	0.16825	23
MOL007777	Stigmasta-5,22-dien-3-O-beta-D-glucopyranoside	21.31817387	0.62593	24
MOL008647	Moupinamide	86.71215907	0.26454	25
MOL011169	Peroxyergosterol	44.39151838	0.82	26

**Table 2:** Top 10 hub targets' PPI network characteristics.

Node_name	Degree	Closeness	Betweenness
IL-6	85	98.5	449.21539
ALB	85	98.5	931.38969
AKT1	82	97	554.8591
TNF	82	97	336.94745
INS	82	97	793.32966
VEGFA	79	95.5	291.65668
MAPK3	72	92	165.21741
IL-10	69	90.33333	122.36172
CXCL8	68	89.83333	141.47683
CCL2	66	88.66667	80.77667

**Table 3:** Analysis of KEGG pathway enrichment using the XF-CRS network (top 20 with count).

Pathway	Enrichment score	P value	Count	Genes
Inflammatory bowel disease (IBD)	9.884173094	3.08*E* − 16	17	IL-10, JUN, TGFB1, SMAD3, STAT1, IL-13, STAT3, TNF, IL-2, RELA, NFKB1, IL-4, IL-6, IFNG, IL-1B, TLR4, IL-17 A
HIF-1 signaling pathway	3.721318074	8.99*E* − 16	19	NOS2, NOS3, EGF, STAT3, PRKCA, IGF1, HIF1A, EGFR, RELA, NFKB1, INS, VEGFA, IL-6, IFNG, BCL2, AKT1, HMOX1, TLR4, MAPK3
Chagas disease (American trypanosomiasis)	9.884173094	4.18*E* − 15	19	IL-10, JUN, TGFB1, CXCL8, SMAD3, NOS2, TNF, IL-2, RELA, NFKB1, IL-6, MAPK8, IFNG, IL-1B, CCL5, CCL2, AKT1, TLR4, MAPK3
Influenza A	9.884173094	3.59*E* − 13	21	PRSS1, JUN, CXCL8, IFNA1, STAT1, PRKCA, PLG, TNF, RELA, NFKB1, ICAM1, IL-6, MAPK8, IFNG, IL-1B, CCL5, CCL2, AKT1, NLRP3, TLR4, MAPK3
Leishmaniasis	9.884173094	9.03*E* − 13	15	IL-10, JUN, TGFB1, ITGAM, NOS2, STAT1, PTGS2, TNF, RELA, NFKB1, IL-4, IFNG, IL-1B, TLR4, MAPK3
Amoebiasis	9.884173094	1.47*E* − 12	17	IL-10, ARG2, TGFB1, ITGAM, CXCL8, CSF2, NOS2, HSPB1, PRKCA, TNF, RELA, NFKB1, IL-6, IFNG, IL-1B, CASP3, TLR4
TNF signaling pathway	9.884173094	1.71*E* – 12	17	JUN, VCAM1, CSF2, PTGS2, TNF, MMP9, RELA, NFKB1, ICAM1, IL-6, MAPK8, IL-1B, CASP3, CCL5, CCL2, AKT1, MAPK3
Pertussis	9.884173094	2.01*E* − 12	15	IL-10, JUN, ITGAM, CXCL8, NOS2, TNF, RELA, NFKB1, IL-6, MAPK8, IL-1B, CASP3, NLRP3, TLR4, MAPK3
Toxoplasmosis	9.884173094	2.66*E* − 12	17	IL-10, TGFB1, NOS2, STAT1, STAT3, TNF, RELA, NFKB1, MAPK8, CD40LG, IFNG, CASP3, ALOX5, BCL2, AKT1, TLR4, MAPK3
Tuberculosis	9.884173094	5.19*E* − 12	20	IL-10, TGFB1, ITGAM, NOS2, IFNA1, STAT1, SRC, TNF, RELA, NFKB1, IL-6, MAPK8, IFNG, IL-1B, CASP3, BCL2, BAX, AKT1, TLR4, MAPK3
Toll-like receptor signaling pathway	9.884173094	2.62*E* − 10	15	JUN, CXCL8, IFNA1, STAT1, CD80, TNF, RELA, NFKB1, IL-6, MAPK8, IL-1B, CCL5, AKT1, TLR4, MAPK3
NOD-like receptor signaling pathway	9.884173094	3.03*E* − 10	12	IL-6, HSP90AA1, MAPK8, CXCL8, CCL5, IL-1B, NLRP3, CCL2, TNF, RELA, NFKB1, MAPK3
Pancreatic cancer	3.592461238	1.64*E* − 09	12	MAPK8, TGFB1, SMAD3, STAT1, EGF, STAT3, AKT1, RELA, EGFR, NFKB1, MAPK3, VEGFA
*Salmonella* infection	9.884173094	2.38*E* − 08	12	IL-6, JUN, MAPK8, CXCL8, CSF2, IFNG, NOS2, IL-1B, TLR4, RELA, NFKB1, MAPK3
Non-alcoholic fatty liver disease (NAFLD)	9.884173094	2.87*E* − 08	15	XBP1, JUN, TGFB1, CXCL8, TNF, RELA, NFKB1, INS, ERN1, IL-6, MAPK8, IL-1B, CASP3, BAX, AKT1
Legionellosis	9.884173094	6.24*E* − 08	10	IL-6, VCP, ITGAM, CXCL8, IL-1B, CASP3, TNF, TLR4, RELA, NFKB1
PI3K-Akt signaling pathway	3.721318074	7.90*E* − 08	21	CSF3, HSP90AA1, IFNA1, NOS3, EGF, PRKCA, IGF1, EGFR, IL-2, RELA, NFKB1, INS, VEGFA, IL-4, IL-3, FGF7, IL-6, BCL2, AKT1, TLR4, MAPK3
Osteoclast differentiation	3.592461238	3.47*E* − 07	13	JUN, TGFB1, STAT1, TNF, RELA, NFKB1, MAPK8, IFNG, IL-1B, BTK, AKT1, PPARG, MAPK3
Prostate cancer	3.721318074	4.72*E* − 07	11	HSP90AA1, EGF, BCL2, CTNNB1, AKT1, IGF1, RELA, EGFR, NFKB1, MAPK3, INS
Hepatitis C	3.592461238	3.05*E* − 06	12	MAPK8, CXCL8, IFNA1, STAT1, EGF, STAT3, AKT1, TNF, RELA, EGFR, NFKB1, MAPK3

## Data Availability

The data used to support the findings of this study are included in the article.
